# Policymakers’ and healthcare providers’ perspectives on the introduction of oral pre-exposure prophylaxis for key populations in Ghana

**DOI:** 10.1186/s12889-023-15871-w

**Published:** 2023-06-05

**Authors:** Henry Nagai, Edward Adiibokah, Henry Tagoe, Waimar Tun, Nanlesta A. Pilgrim, Augustine Ankomah, Yussif Ahmed Abdul Rahman, Stephen Ayisi Addo, Stephen Kyeremeh Atuahene, Emmanuel Essandoh, Sean Maher, Mark Kowalski

**Affiliations:** 1 JSI Research & Training Institute, Inc, Accra, Ghana; 2 Population Council, Accra, Ghana; 3 Population Council, Washington, DC USA; 4 National AIDS/STI Control Programme, Accra, Ghana; 5 Ghana AIDS Commission, Accra, Ghana; 6 United States Agency for International Development, Accra, Ghana; 7 JSI Research & Training Institute, Inc, Boston, USA

**Keywords:** Policymakers, Healthcare providers, Pre-Exposure Prophylaxis (PrEP), Men who have sex with men, Female sex workers, HIV, Ghana

## Abstract

**Background:**

Key populations (KPs) such as female sex workers (FSWs), men who have sex with men (MSM), people who inject drugs (PWID), and their partners contribute more than a quarter (27.5%) of new HIV infection in Ghana. Oral pre-exposure prophylaxis (PrEP) can substantially reduce HIV acquisition among this group. While the available research indicates KPs willingness to take PrEP in Ghana, little is known about the position of policymakers and healthcare providers on the introduction of PrEP for KPs.

**Methods:**

Qualitative data were collected from September to October 2017 in the Greater Accra (GA) and Brong-Ahafo (BA) regions of Ghana. Key informant interviews were conducted with 20 regional and national policymakers and supplemented with In-depth Interviews with 23 healthcare providers to explore their level of support for PrEP and their perspectives on challenges and issues to consider for oral PrEP implementation in Ghana. Thematic content analysis was used to unearth the issues emerging from the interviews.

**Results:**

Policymakers and healthcare providers in both regions expressed strong support for introducing PrEP for KPs. Key concerns regarding oral PrEP introduction included potential for behavioral disinhibition, non-adherence and side effects of medication, cost and long-term financial implications, and stigma related to HIV and key populations. Participants stressed the need to integrate PrEP into existing services and the provision of PrEP should start with high risk groups like sero-discordant couples, FSWs and MSM.

**Conclusions:**

Policymakers and providers recognize the value of PrEP in cubing new HIV infections but have concerns about disinhibition, non-adherence, and cost. Therefore, the Ghana health service should roll-out a range of strategies to address their concerns including: sensitization with providers to mitigate underlying stigma towards KPs, particularly MSM, integration of PrEP into existing services, and innovative strategies to improve continued use of PrEP.

## Introduction

The adoption and scale-up of pre-exposure prophylaxis (PrEP) for HIV is key for the global target of ending new HIV infection by 2030 [1, 2]. Yet there is low uptake in countries of sub-Saharan Africa (SSA) that need it most [3]. As noted by the World Health Organization in 2015, PrEP is recommended for groups at substantial risk of HIV infection [4]. This is particularly so, for countries like Ghana with a low-level HIV epidemic of less than 2% over the last decade but high HIV prevalence among key populations (KP) at risk for HIV such as men who have sex with men (MSM), female sex workers (FSWs), transgender individuals, and their partners. A high proportion of new HIV infections in Ghana (28%) occurs among these high-risk populations [5, 6].

Ghana is one of the few countries in SSA that has participated in a PrEP efficacy and safety trial [7, 8] Between June 2004 and March 2006, 400 Ghanaian women took part in a randomized, double-blind, placebo-controlled study, where they took daily oral tenofovir disoproxil fumarate (TDF) over a 12-month period. Adherence was greater than 82% throughout the trial period [8]. Contrary to concerns regarding behavioral disinhibition, the trial noted decreases in high risk behaviors among participants including reduction in sexual partners, reduction in rates of unprotected sex acts, and positive changes in pregnancy prevention practices [8]. In 2012, Adedotun Ogunbajo et al., explored the knowledge and acceptability of HIV PrEP among Ghanaian MSM in Accra, Kumasi, and Manya Krobo. The qualitative study which involved 22 focus group discussions with MSM participants, revealed that though participants had low knowledge of PrEP, once information about PrEP was provided to them, there was high acceptability [9]. While the acceptability of PrEP by end-users, especially high risk groups like MSM and FSW, is well documented, the views of policymakers and service providers has not been well researched[10–14]. Understanding policy makers’ awareness, attitudes, perceptions, and preferences around a health product such as PrEP is very important in guiding its introduction and roll-out [15]. Literature suggests that eliciting policymakers or policy influencers opinions to help shape health care interventions such as PrEP, increases the likelihood of the utilization of research evidence by policymakers as well as the success of such interventions [16].

Two years after the WHO recommendation for PrEP to be adapted for HIV prevention among high-risk groups, Ghana had yet to include PrEP into its HIV combination prevention framework as of 2017; policymakers and other key stakeholders remained hesitant around the introduction of PrEP, due to the paucity of context-specific research to inform Ghana’s decisions on PrEP introduction [17]. This article interrogates how policymakers in Ghana perceive the introduction of PrEP for KPs and what would be needed to support PrEP introduction and scale-up in Ghana.

### METHOD

Qualitative data were collected from September-October 2017 in the Greater Accra (GA) and Brong-Ahafo (BA) regions of Ghana. These two regions represent two of the then ten regions of Ghana, and they were selected to represent the southern and northern zones, respectively, with GA being the most urbanized and cosmopolitan region in the country. The BA region lies in the middle belt of the country and is considered a transitional zone attracting populations from both northern and southern sections of the country. After the completion of the study, BA region was divided into three separate regions – Bono, Bono East, and Ahafo. Most study participants came from what is now considered the Bono region [15].

Key informant interviews (KII) were conducted with 20 national and regional policymakers (9 from GA and 11 from BA) in the area of HIV and AIDS policy and programming. Experts were purposefully selected from Ghana Health Service, National AIDS/STI Control Program (NACP), Ghana AIDS Commission (GAC), and Regional/District HIV Coordinators from Greater Accra and Brong-Ahafo regions. These are institutions whose activities directly determine and influence HIV programs and health policy. Participants had to be a national or regional director or manager working with any of these nationally established HIV /AIDS bodies [15]. This was complimented with in-depth interviews with 23 service providers. To ensure heterogeneity and diversity, the research team purposively selected providers to cover factors such as the type of facility ownership (e.g., private, public, non-profit), facility size, the four different cadres (doctors/nurses, pharmacists/dispensing technicians, counselors, and case managers/peer educators). For the in-depth interviews with the providers, the research team purposefully selected providers based on being part of senior management of the facility and experience in providing HIV or SRH services to patients. The total number of interviews conducted was guided by theoretical saturation.

Semi-structured interview guides were developed by the authors and were field-tested to ensure that questions were appropriate for the intended respondents. Interviews with policymakers explored PrEP perceptions and steps needed to facilitate PrEP introduction in Ghana. Interviews with providers elicited information about feasibility, acceptability, and barriers and facilitators associated with PrEP introduction. Ten [10] trained qualitative researchers, with at least a First Degree in Social Science and prior experience in conducting qualitative interviews and key population (KP) research were recruited to conduct the interview. They were recruited after one week of qualitative research and interview skills training. The interviews were conducted in English that lasted between 45 min to one hour. The interviews were supervised by two members of the research team and co-authors (HT and EA). To ensure the research team had a comprehensive understanding of the issues faced by Key Populations in Ghana, they received key population competency training as part of their training. In addition, they were encouraged to keep a reflexivity journal to capture and reflect on any insights that emerged during data collection, processing and the analysis of the results [18]. To ensure that participants feel free to express themselves on issues, interviews were conducted in a safe, private and confidential locations or at the workplace of the participants. Consent were sought from participants before interview to digitally record and transcribed verbatim for accuracy, credibility and reliability of information.

A critical consideration by the research team is the subject of positionality and reflexivity due to the sensitivity around issues of key populations and the levels of stigma and discrimination KPs are subject to in Ghana. Through value-clarification, consensuses building among the research team members, the team were able to acknowledged, reconsider and mitigate any pre-existing positions, biases and assumptions on the subject of KP and PrEP. Validation of the preliminary results with major stakeholders including KP communities and policymakers in Ghana, allowed and ensure that the results were consistent with the experiences and perspectives of those most directly affected by the subject under study.

### Data Analysis

Five out of 20 transcripts were sampled across the different stratum (2 national and 3 regional) and two of each cadre of providers given to the team of researchers to read and develop codes. The codes generated were compared for inter-coder reliability and discussed in relation to the study objectives and the research questions that underpinned the study and consensus was reached among the research team through an iterative process. The categories and sub-categories emerging from the transcripts were finalized into a codebook with clear definitions. The codebook and database with transcripts were entered into a qualitative analysis software package (QSR NVivo 11) [15]. Two trained qualitative research assistants coded the transcript. The research team (HT, EA, and WT) processed the coded data by running different queries along the main categories (individual and structural challenges and facilitators) using a qualitative content analysis approach [19, 20]. Conscious efforts were taken by research team to ensure no influence of personal biases in the development of codes, analysis, triangulating between KIIs and IDIs data for credibility and the degree of neutrality in the research study’s findings for confirmability [21, 22].

## Results

Of the 20 policymakers interviewed for the KIIs, six were national policymakers from Accra and 14 were regional policymakers (7 from GA and 7 from BA). The subset of 23 providers (14 from GA and 11 from BA) who participated in the IDIs constituted five nurses, five doctors/physician assistants, five pharmacists/dispensing technicians, and eight counselors/case managers/peer educators.

The results are organized along three major theoretical themes: (i) Support for and perceived benefits of PrEP; (ii) Concerns to the integration of PrEP in programming, and iii. Key issues to consider for the successful introduction of PrEP.

### Support for and perceived benefits of PrEP

Overall, most policymakers and providers expressed support for PrEP as an HIV prevention intervention and expressed willingness to advocate for its use, especially among KPs. Two subthemes emerged as reasons for their support of the strategy: (i) PrEP could be an appropriate and effective strategy for mitigating HIV infection among KPs; and (ii) PrEP complements condoms and other prevention strategies.

“I am so happy that we have taken this initiative and I think it will go a long way for us… Let’s initiate it, let’s do more advocacy than implementation now and see the way forward to help introduce PrEP.” (National level policymaker)

“I don’t see any problem with prescribing PrEP …Our main aim is to reduce HIV and so if something is there to reduce the risk, why not?” (Provider, Brong Ahafo)

A few service providers were, however, opposed to giving PrEP to AGYW – especially those around the age of 15, because they thought it would promote promiscuity.

“……below 18 I will not feel so comfortable giving it to them. Below 18 years they are educated in school, once they start menstruating, they are educated and told that if they start having sex recklessly, they may get infected with HIV. From 18 years they can be educated on this medicine, and it can be given to them. (Provider Greater Accra)

Many policymakers and providers saw PrEP as a complement to condoms as an extra method of protection for KPs, given that condoms generally are not consistently used in these high-risk groups.

“You know some of the key population groups, especially the FSW, some of them sometimes take more money to have sex without condoms, or the condom may burst. So, if they have something to protect them from HIV that is good” (Regional level policymaker)

“Everyone has a choice so when PrEP is introduced, some may use PrEP and others may use condoms, so I think the two in the system will help. …It is important to use both because taking PrEP protects you from getting HIV but using the condom protects you from other STIs as well.” (Provider, Brong-Ahafo)

### Concerns for the integration of PrEP for KP programming

While the majority were in favor of PrEP introduction in Ghana, there were various concerns expressed about PrEP. Four subthemes emerged as key concerns about PrEP introduction: (i) Potential for behavioral disinhibition; (ii) Non-adherence and side effects of medication; (iii) Cost and long-term financial implications; and (iv) Perceived stigma.

i) Potential for behavioral disinhibition.

Some policymakers and providers expressed concern that introducing PrEP would lead to riskier sexual behaviors among KPs, and therefore, increase the transmission of other sexually transmitted infections and rates of unintended pregnancies.

“The concerns could also be many, because what if people abuse it? The fact that there’s a preventive medication, it will allow some people to live careless lives, to have unprotected sex because they think there’s a medication to prevent them from getting HIV.” (Regional level policymaker).

“Once we say that take it, you won’t get it, and it is this effective then it means they are going to open the floodgates, and everybody will be doing it [referring to sex] without any caution… It doesn’t protect against STIs. So, if we are going to do that, HIV is the one that everybody is afraid of. For pregnancies, we will be getting a lot of teenage pregnancies, we will be getting a lot of gonorrhea and all the other STIs and it is going to be a problem, so that’s my concern.” (Provider, Brong-Ahafo).

ii) Non-adherence and side effects of medication.

Some policymakers and providers expressed concern about PrEP adherence as a possible introduction barrier. In particular, a key concern was PrEP’s possible side effects and their influence on adherence. Providers explained that side effects can lead to non-adherence and subsequently lead to drug resistance. As a result, policymakers expressed interest in more information about PrEP’s long-term effects, particularly what sustained and continued use might do to individuals’ overall health.

“But for the side effect, that is my worry... the unknown side effects of the medication and its implication to the person’s health…” (National level Policymaker,)

“The side effects I am already concerned about because since the person hasn’t taken the drug s/he is moving around smoothly but maybe the person will start and s/he might experience some minor side effects. S/he will not complete the treatment, will stop and maybe it will be a resistance against the drug.” (Provider, Brong-Ahafo)

iii) Cost and long-term financial implications.

Additionally, policymakers and providers discussed PrEP cost as a potential barrier to adherence. They suggested in-country PrEP production, PrEP inclusion in the national health insurance, and implementation into a fee-system as ways to lower the cost. Ultimately, they felt that the question of financing PrEP would play an instrumental role in KPs’ abilities to access and adhere to the drug.

“So financing, I will be of the view that from the inception we shouldn’t go all free. The beneficiary must contribute something small, even if it is GHs 1 a month.” (Policymaker, National level)

“The cost will actually let us know [about PrEP use]; though they may accept it, but they can’t afford it. So, the government should support the cost of the drug.” (Provider, Greater Accra)

“My main issue is how do we ensure adequate financing of the current HIV prevention program and PrEP as well as a plan for sustainability, such as in-country production of PrEP and a fee-system. So financing, I will be of the view that from the inception we shouldn’t go all free. The beneficiary must contribute something small, (National level policymaker,)

They feared that if PrEP is introduced in Ghana, it will shift resources away from what they consider as their priority, such as prevention of mother-to child transmission.

“You don’t have money for your child’s school fees, yet you are looking for money to celebrate birthday for him. In this country we don’t have enough ARVs for those who are sick already, PMTCT is a problem, and you want to give medication to those who are not sick” (National level policymaker).

iv) Perceived stigma.

Perceived stigma heavily influenced policymaker’s and providers’ views of PrEP. They spoke about various forms of stigma -- stigma related to HIV and taking medications associated with HIV as well as stigma associated with sex work, promiscuity, and same-sex sexual behaviors. Providers expressed concern about how clients would negotiate that stigma in a service setting and how providers can foster an environment that does not magnify stigma. Policymakers and providers indicated that stigma could then deter KPs and others from taking PrEP.

“Stigma and discrimination will come at the point of maybe, let say a young girl is going to buy …the drug. The PrEP at the pharmacy. …So, imagine this girl knows she can take some medication to take to prevent herself from getting HIV. Then she comes to the pharmacy and then maybe an elderly person who sees her and knows the use of the pre-exposure prophylaxis. So, stigma may come still at the community level, so we have to address the stigma… It has to change the people’s perception towards sex.” (Regional level policymaker)

### Key issues to consider for successful PrEP introduction

Policymakers and providers made a number of recommendations for the effective implementation of PrEP in Ghana. Four subthemes emerged: (i) Community education and sensitization; (ii) adequate preparation of health services to provide PrEP, including addressing stigma among healthcare providers; (iii) PrEP integration into health services; and (iv) more implementation science research.

i) Community education and sensitization.

Most policymakers emphasized the need for a robust community-wide outreach and educational campaign for PrEP introduction to succeed. Additionally, participants highlighted the need for a PrEP campaign not solely focusing on educating high-risk groups about PrEP, but also reaching the broader community to help build community-wide support for PrEP as an HIV prevention method and reduce misinformation and stigma.

“Maybe we give about six months’ continuous education so that we know that almost 98% of the populace have been covered with what the PrEP does, the side effects…and then what we should expect in the next four/five years of taking PrEP.” (Regional level policymaker).

ii) Adequate preparation of health services to provide PrEP, including addressing stigma among healthcare providers.

Policymakers and providers also proposed strategies to adequately prepare the health care system and providers to provide high quality PrEP care. Participants spoke about the need to not only train on the clinical aspect of PrEP provision, but also on stigma reduction and boosting health providers’ ability to provide client-friendly care, particularly to populations that have traditionally been stigmatized in healthcare settings, such as MSM and FSWs.

“We have to educate the health workers because the stigmatization is not only for those outside… you have to give the staff some kind of training to know that we should accept the MSMs and the FSWs” (Provider, Brong-Ahafo).

iii) PrEP integration into health services.

Both policymakers and providers voiced support for PrEP integration into health services beyond HIV, such as family planning (FP) and general health services. Majority of policymakers and service providers advanced the notion that PrEP service provision should not be isolated or silo but should be integrated into health service settings where the following services are offered: HIV counseling and testing, FP and reproductive health, STI services, and maternal health settings. While they felt integration was important, they indicated concern that the addition of PrEP to existing services will increase the workload and require additional staff due to potential increase in clients and the amount of time necessary to provide the counseling needed for PrEP. As a consequence, they feared the quality of services could decrease with this increased workload.

“I’m advocating and recommending that though [PrEP is] important, its integration into the current program activities and the intervention should be looked at carefully.” (Regional level policymaker).

“It should be combined with other things, like maybe family planning [or] child welfare.” (Provider, Greater Accra).

“Human resources because it becomes like the same person doing all the normal work including HIV, TB and now we are coming to offer a whole new service that you need to even take time with adherence because it is new, yes so I think to me that will be my challenge.” (Provider, Greater Accra).

iv) More implementation science research.

Policymakers requested more implementation science research to uncover and address the real-world challenges of PrEP implementation. They also discussed the need to adapt existing strategies from other countries and conduct case studies to learn from other African countries that are rolling out PrEP.

“If we can look at example countries that have already implemented them, we can look at them as our role models, look at the benefits and disadvantages, what they have gained so far” (Regional level policymaker).

## Discussion

To the best of our knowledge this is the first study in Ghana assessing perceptions of policymakers and healthcare providers regarding PrEP introduction in Ghana. Overall, policymakers recognized the value of PrEP but had concerns about behavioral disinhibition, non-adherence, financial costs, and stigma. Although, Ghana has rolled out oral PrEP for key populations and other high-risk persons such as adolescents and young men and women and negative partners of HIV positive persons since 2020 [23], it is important to document and share these key lessons to inform the Ghanaian context when new delivery methods of PrEP are available (e.g., long acting injectable, dapivirine vaginal ring). The results of this study will also be useful for the introduction of similar health products for at-risk populations.

As emphasized by both policymakers and providers, integration of PrEP into existing services will be key to the successful implementation of PrEP. This finding, in fact, echoes what is in the WHO guidelines [24] for PrEP implementation, which recommends PrEP be integrated into existing health services such as HIV testing services and ART as these services already have the resources (e.g., staff, infrastructure, HIV testing, and medications) necessary to offer PrEP. The guidelines also suggest considering integrating PrEP into other settings such as sexual health clinics, FP services, and service settings for key populations. Integration of PrEP into public HIV care clinics has been found to be feasible in African countries. A recent large scale cluster randomized trial of PrEP integration into 25 HIV care clinics in Kenya found high PrEP uptake with reasonable level of continuation with high adherence, and low HIV incidence [25]. Policymakers in this study also mentioned PrEP integration into existing services outside of HIV services, for example FP services. The 2019 ECHO trial results, which showed high HIV incidence among FP clients reinvigorated calls to integrate HIV prevention services (including PrEP) into FP [26]. There is a growing number of demonstration projects of integrating PrEP into various sexual and reproductive health services in sub-Saharan Africa with evidence of feasibility and acceptability [27–32]. Integration models range from a provider providing a range of FP and HIV services to one facility providing a range of FP and HIV services with strong referral mechanisms [33]. Bhavaraju et al. (2021) outlined elements spanning five health system domains (plans and policies, resource management, service delivery, PrEP use, and monitoring and reporting) for effective PrEP-FP integration [33]. While policymakers felt strongly that PrEP should be integrated into existing health services, there was less enthusiasm for PrEP to be integrated into services offered outside the typical clinical platforms/settings such as through non-governmental organizations, mobile health services, and faith-based organizations. This may present a programmatic challenge particularly in light of the fact that the populations at high risk for HIV such as MSM, FSWs and adolescents (for whom PrEP is recommended) are effectively reached through community-based or grassroots organizations. Therefore, there will need to be close collaboration between health facilities offering PrEP and community-based organizations that can effectively mobilize and create demand among high-risk populations.

Service providers and policymakers also expressed concerns that introducing PrEP would lead to KPs engaging in riskier sexual behaviours and therefore, increase the transmission of other sexually transmitted infections and rates of unintended pregnancies. Recent studies and reviews, including those in sub-Saharan African, found minimal to no evidence of risk compensation [34–38]. Despite this, health experts caution that although risk compensation is not likely to reduce the benefit of PrEP, clinicians should continue to recommend PrEP as a supplement to condoms rather than a replacement for them.

Policymakers’ concerns about financial implications of PrEP emanated from two main standpoints. The first was concerns that PrEP services would take away scarce donor funds from critically needed antiretroviral drugs for those already living with HIV. This concern emanated from the realities in Ghana, which often experiences shortages of antiretroviral drugs for people who are already infected with HIV [39, 40]. This underscores the need to address the very realistic concern of policymakers and find strategies to integrate PrEP programming into the overall HIV prevention response in the country. The other financial concern was from the perspective of potential PrEP users. They indicated that even though PrEP was offered free of charge, the costs associated with PrEP use (e.g., laboratory fees, testing for sexually transmitted infections, travel-related expenses) would still be difficult for many of them [41, 42]. Policies should, therefore, critically consider the cost and sustainability issues, as these could negatively impact access to and continued use of oral PrEP. In fact, financial difficulties of PrEP users have been found to be associated with poor HIV protection from PrEP among MSM in West Africa [43].

Both policymakers and providers raised concerns around potential for poor adherence to PrEP. For PrEP to be effective, consistent use among persons at continued risk is critical [44, 45]. This issue has, in fact, been a challenge that has been observed in many oral PrEP programs, particularly in low- and middle-income countries [46]. A global systematic review of adherence to oral PrEP identified a myriad of reasons for poor adherence such as stigma, low risk perception, unacceptable dosing regimens, logistics of daily life, and side effects [46]. As such, it will be critical for PrEP services to be implemented as part of a multi-faceted comprehensive program that addresses these barriers. Further, this also points to the pressing need for long-acting injectable PrEP and other delivery methods as an alternative to the daily oral PrEP.

Lastly, this study highlighted how stigma acts as a major barrier to PrEP uptake and that without addressing stigma, the uptake of oral PrEP may be slow, particularly in mainstream facilities. This is in line with a large body of evidence, including from sub-Saharan Africa, indicating PrEP-related stigma as a key barrier to PrEP uptake, in particular, fear of being regarded as engaging in high-risk activity (promiscuity) or being HIV-positive if they take PrEP [11, 47–53].This study also highlighted that providers are not comfortable providing services to certain high-risk populations such as MSM. Given the high levels of stigma associated with HIV as well as with same-sex sexual behaviors, HIV programs will need to sensitize and train both clinical and non-clinical staff at mainstream facilities to ensure a safe and enabling environment for all type of clients.

The main limitation of this study is that opinions of providers regarding their willingness to provide PrEP were based on a hypothetical situation at the time they responded as PrEP had not been available at the time of the study. Therefore, responses may have been limited by their lack of sufficient information about oral PrEP. Future studies should assess actual provision of oral PrEP among providers now that it has become more widely available.

## Conclusion

This study has shown that while there is a high level of support for the introduction and scale-up of oral PrEP, there is a need for the Ghana Health Service, to address various issues related to costs/pricing, HIV and PrEP -related stigma, and utilize strategies to integrate PrEP into general health and sexual and reproductive health services. Robust outreach and education campaign at the community level is also needed to ensure that all levels of society have access to accurate information on PrEP use, adherence, and effectiveness.

## Data Availability

The datasets generated and/or analyzed during the current study are not publicly available due the sensitivity and position of participants involved in the study but are available from the corresponding author on reasonable request.

## Abbreviations

AIDS: Acquired immunodeficiency syndrome
ART: Anti-retroviral therapy
BA: Brong-Ahafo regions
Care Continuum: The USAID Strengthening the Care Continuum Project
CHAG: Christian Health Association of Ghana
CHPS: Community-based Health Planning and Services
FGD: Focus group discussion
FSW: Female sex worker
GAC: Ghana AIDS Commission
GDHS: Ghana Demographic and Health Survey
GA: Greater Accra
GoG: Government of Ghana
GNSP: Ghana National HIV and AIDs Strategic Plan
HIV: Human immunodeficiency virus
IBBSS: Integrated bio-behavioral surveillance survey
ICF: Informed consent form
JSI: JSI Research & Training Institute, Inc.
KII: Key informant interview
KP: Key population
LNGO: Local non-governmental organization
MOH: Ministry of Health
MSM: Men who have sex with men
NACP: National AIDS/STI Control Programme
PC: Population Council
PI: Principal investigators
PLHIV: Persons living with HIV
PrEP: Pre-exposure prophylaxis
SSA: sub-Saharan Africa
SRH: Sexual and reproductive health
USAID: United States Agency for International Development
TDF: Tenofovir disoproxil fumarate
